# Enhanced Reaction Time Measurement System Based on 3D Accelerometer in Athletics

**DOI:** 10.3390/s25216730

**Published:** 2025-11-03

**Authors:** Antonio Pousibet-Garrido, Juan A. Moreno-Pérez, Pablo Escobedo, Israel Caraballo, José V. Gutiérrez-Manzanedo, José L. González-Montesinos, Miguel A. Carvajal

**Affiliations:** 1 PRIMELab, ECsens, Sport and Health University Research Institute (iMUDS), Department of Electronics and Computer Technology, Research Centre for Information and Communications Technologies (CITIC), University of Granada, 18071 Granada, Spain; antoniopoug@ugr.es (A.P.-G.); juanantoniomp@ugr.es (J.A.M.-P.); pabloescobedo@ugr.es (P.E.); 2 Department of Physical Education, Faculty of Education Sciences, University of Cádiz, 11519 Cádiz, Spain; israel.caraballo@uca.es (I.C.); jgmontesinos@uca.es (J.L.G.-M.)

**Keywords:** reaction time, inertial measurement unit (IMU), athletics

## Abstract

**Highlights:**

**What are the main findings?**

- A portable system for measuring reaction time at athletic starts was designed and developed using an Inertial Measurement Unit (IMU).
- Validation tests demonstrated good agreement with the high-speed camera, used as the gold-standard reference system.

**What is the implication of the main finding?**

- The proposed portable, low-cost system provides reliable reaction time measurements in athletics, offering valuable support for athletes and coaches.

**Abstract:**

Reaction time (RT) is a key measure of neuromuscular and cognitive performance, but most existing methods rely on laboratory equipment or focus on localized actions such as finger taps or foot lifts, limiting their relevance for whole-body movements. In this work, we present a portable inertial measurement unit (IMU)-based system specifically designed to measure RT during sprint starts. The device integrates a triaxial accelerometer (ICM-20948, ±16 g) and an ESP32 microcontroller, which generates an auditory stimulus, acquires acceleration data at 1 kHz, and computes movement onset in real time. A fixed acceleration threshold, determined from calibration against a high-speed camera reference, was used to detect the first voluntary movement. Both desktop and smartphone applications were implemented to control the system, provide feedback, and store test data. Validation experiments showed good agreement with the high-speed camera used as a reference (R^2^ = 0.9391), with a mean bias of –1.35 ms and 95% limits of agreement within ±25 ms. The proposed system combines high temporal resolution, portability, and straightforward deployment, enabling reliable assessment of whole-body RT in realistic sports and research environments.

## 1. Introduction

Reaction time (RT) measurement is a fundamental metric for evaluating neuromuscular function, cognitive performance, neurological health, and overall human motor responsiveness [1,2]. This temporal metric has proven valuable across diverse domains, including clinical diagnostics, sports performance, and occupational safety [3]. In clinical settings, reaction time measurements have demonstrated utility in assessing age-related cognitive decline and neurological conditions [4], screening for conditions such as mild traumatic brain injury [5], and even monitoring alcohol-induced impairments [6]. In the context of workplace safety and occupational health, reaction time is often used to evaluate alertness and cognitive efficiency in high-risk jobs [7,8]. In sports science, the reaction time metric offers insights into an athlete’s sprint start performance, fatigue levels, and overall neuromuscular responsiveness [9,10], providing researchers and coaches with valuable real-time feedback capabilities. For instance, some studies have highlighted that sprint acceleration involves rapid postural and kinematic changes that demand precise motor timing and fast neuromuscular coordination, characteristics that can be sensitively captured by reaction time measurements [11]. Another study reported that thigh angular acceleration during the swing phase of steady-speed running correlates with sprint performance and can be accurately captured via wearable sensors [12].

Traditional reaction time assessment methods, such as camera-based motion capture systems or desktop-based tests, while accurate, are often limited by their cost, immobility, and complexity, restricting their application to controlled laboratory environments [13,14,15]. These limitations hinder field deployment for continuous monitoring in real-life scenarios [5]. To overcome these barriers, inertial measurement units (IMUs) have emerged as a promising alternative for reaction time assessment and, in general, human motion analysis [16]. IMUs, typically composed of accelerometers, gyroscopes, and magnetometers, enable real-time, wireless tracking of motion and have gained popularity due to their affordability, portability, and ease of integration into wearable platforms [17,18].

The evolution of IMU-based reaction time measurement systems has progressed through various technological approaches, from early single-sensor setups using only accelerometers to capture discrete motion events [19,20,21,22], to more advanced multi-sensor configurations that enhance temporal precision and motion characterization [5,23,24]. For example, in clinical research, accelerometer-based sit-to-stand measurements have been validated for reliability in older adults, highlighting its functional applicability and concurrent validity against motion capture systems [25]. Large-scale reviews have also confirmed the general accuracy of accelerometer-based assessments in real-world settings, including child physical behavior contexts [3].

Validation efforts have shown promising agreement between IMU-based reaction time assessments and traditional gold-standard techniques. For example, stride-frequency and thigh acceleration metrics derived from IMUs showed strong validity in dynamic sprint tasks, with sensor-based measurements closely aligning with motion capture data [12,26]. Similarly, comparison of accelerometer-only systems against 3D motion analysis demonstrated concurrent validity in lower-limb motor reaction timings [27]. Multi-sensor IMU setups incorporating gyroscopes and magnetometers have further improved event detection reliability [23,24]. Furthermore, recent technological advances have expanded the scope of IMU-based reaction time assessment beyond basic locomotor tasks. Smartphone-based implementations have democratized access [28,29], while integration with virtual reality environments has introduced novel forms of cognitive-motor interaction for training and evaluation [30]. These developments have the potential to significantly enhance athlete engagement, remote diagnostics, and immersive neuromechanical testing.

However, current research also highlights several challenges in IMU-based reaction time measurement. IMU signal accuracy can vary significantly depending on sensor placement and attachment method, which affects the fidelity of reaction time measurements [31]. Similarly, inadequate sampling rates can compromise the detection of rapid motor events. In fact, recent studies have emphasized the importance of maintaining high temporal resolution (particularly during dynamic activities like sprinting or sudden deceleration) to capture short-latency responses accurately [32]. Finally, the lack of standardized protocols and benchmarks across devices, sensor placements, and event definitions remains a barrier to widespread adoption and cross-study comparability. Despite recent advances in camera-based, smartphone, and IMU-driven approaches, current systems remain limited by low sampling rates, trial-dependent thresholds, or a focus on distal surrogate tasks rather than full-body responses. Our proposed solution addresses these gaps by combining high-frequency acceleration sensing (1 kHz) with synchronized auditory stimulation and fixed-threshold detection, validated against a high-speed camera reference. Unlike previous portable systems, it captures the true onset of whole-body movement during sprint initiation, offering robust, real-time, and field-ready assessment of neuromuscular reaction time. Therefore, our hypothesis is that the developed IMU system allows for reliable measurement of reaction times in sprint starts in athletics. The objectives of the present study were the following:

i. To design and implement an IMU-based system for measuring reaction time in athletics.
ii. To determine athletes’ reaction times to an auditory stimulus during a sprint start, using both a high-speed camera and the proposed IMU system.
iii. To compare the reaction time results obtained from the high-speed camera with those from the IMU system.

## 2. System Description

To implement a field-ready solution, we developed an IMU-based reaction time assessment system building upon the portable and lightweight validated inertial sensing architecture previously introduced for vertical jump analysis [33]. In the original system, a custom-developed IMU-based system mounted near the body’s center of mass was employed to estimate flight time and jump height with high accuracy. The platform utilized a 9DoF Razor IMU (MPU-9250, TDK InvenSense, San Jose, CA, USA), which integrates a triple-axis accelerometer, gyroscope, and magnetometer. The IMU is controlled via a SAMD21 microcontroller (Microchip Technologies Inc., Chandler, AZ, USA) and communicates wirelessly to a custom Android application through a BM78 Bluetooth (BT) module (Microchip Technologies Inc.). The device is powered by a 450 mAh lithium-polymer battery (LiPol Battery Co., Ltd., Shenzhen, China) providing approximately 9 h of continuous operation. Acceleration data were sampled at 200 Hz and transmitted in real time to the user smartphone for on-site analysis, achieving minimal latency and high fidelity.

The current version of the system has been repurposed to assess a different yet equally dynamic scenario: the measurement of reaction time during explosive sprint start actions, with a focus on acceleration-triggered event detection. The system, originally designed to detect jump take-off and landing, was modified at both hardware and firmware levels to identify voluntary movement onset following an auditory cue randomly triggered by the custom-developed smartphone and desktop application. The reaction time was measured as the delay between this auditory stimulus provided by a buzzer and the first detectable acceleration peak exceeding a predefined threshold. Therefore, unlike conventional reaction time setups that rely on finger taps or foot lifts, this protocol captures the full-body response to the auditory external stimulus from a starting block position, enabling a more ecologically valid assessment of neuromuscular responsiveness in sprint and sports contexts.

**Figure 1:**
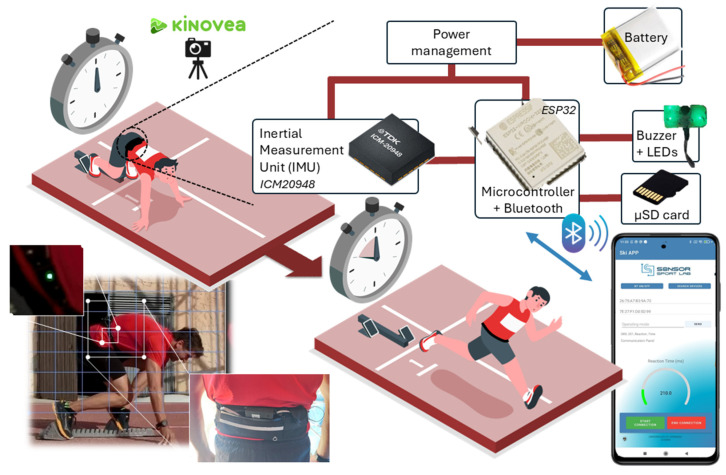
Overview of the developed IMU-based system for reaction time measurement.

The hardware, firmware and Android application have been reconfigured to allow stimulus-triggered data acquisition and onboard real-time computation of motor reaction latency. Acceleration signals are filtered, and movement onset is detected using a threshold-crossing approach. The system is designed to be worn on the lower back of the athlete, as shown in Figure 1, allowing for highly localized reaction time measurements that align with specific neuromuscular pathways. The desktop-based software includes visualization of the acceleration trace and automatic values saving, which are logged alongside timestamps and test conditions. The adaptation of the previously validated platform to reaction time assessment confers several advantages: high temporal resolution (up to 1 kHz), robust motion detection, full portability, and ease of deployment in real-world or athletic environments. Moreover, by leveraging direct acceleration data rather than indirect behavioral cues (e.g., button presses), the system captures true physical movement onset, making it suitable for applications in neuromechanical testing, fatigue assessment, sports performance, and cognitive-motor research.

### 2.1. Hardware Development

**Figure 2:**
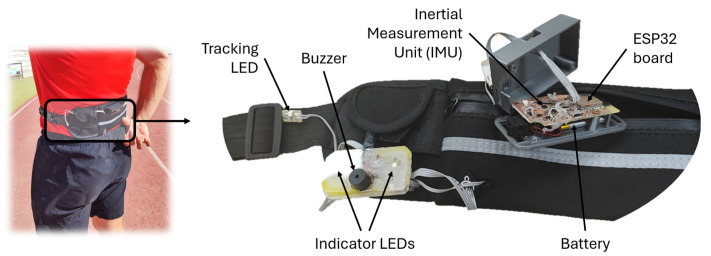
Photograph of the developed system with components labeled.

Figure 2 shows a photograph of the developed measuring system with its main components labeled. The developed system builds upon our previous prototype [33], but this work introduces several significant hardware and software design improvements that substantially enhance performance and functionality. In summary, the system presented in this work incorporates the following novel features and improvements:

- Custom PCB design: An ad hoc PCB was designed and fabricated using an LPKF ProtoMat S100 milling machine (LPKF Laser & Electronics, Garbsen, Germany), integrating all system components (ICM20948 IMU, ESP32 module, power management system, etc.) into a single compact layout. This eliminated the need for commercial development boards and external Bluetooth modules, significantly reducing both size and weight.
- Custom 3D-printed enclosure: A custom PLA case was designed and fabricated using a 3D printer Creality 3D CR-X (Shenzhen Creality 3D Technology Co, Ltd., Shenzhen, China) to precisely fit the custom PCB, replacing the standard commercial enclosure used previously. The device features a compact, wearable, battery-powered design (80 × 40 × 20 mm, 42 g). A 450 mAh battery was selected to maintain the compact form factor constrained by electronic board dimensions while ensuring adequate operational autonomy.
- Upgraded IMU sensor: The new design comprises a 9-degree-of-freedom IMU model ICM20948 (TDK InvenSense, San José, CA, USA), which integrates a three-axis 16-bit accelerometer, with acceleration range from ±2 g to ±16 g and a maximum sample rate of 4.5 kHz, and a three-axis 16-bit gyroscope with a range of ±250 to ±2000 degrees per second (dps). It also incorporates a three-axis 16-bit magnetometer AK09916 (AKM Semiconductor, Inc., Tokyo, Japan) with a range of ±4912 µT. This IMU is an upgraded version of the MPU-9250 9DoF used in the earlier prototype, offering improved performance and lower power consumption. The IMU also integrates a Digital Motion Processor (DMP), which processes the raw sensor data through filtering and sensor fusion to obtain rotation quaternions, later converted into Euler angles (yaw, pitch, and roll). However, even under optimal conditions, the DMP can achieve a maximum frequency of 225 Hz, which is insufficient for high-speed (HS) dynamic measurements [32]. In this work, only the accelerometer data are used, achieving a sampling frequency of 1 kHz for the three acceleration components.
- Enhanced processing capabilities: To enable higher processing power and a more compact implementation, a 32-bit ESP32-WROOM-32E-N16 microcontroller (Espressif Systems Co., Ltd., Shanghai, China) was employed. This MCU features a maximum clock frequency of 240 MHz, 520 kB of RAM, and 16 MB of internal flash memory (3 MB allocated for program storage) and wireless communication capabilities (Wi-Fi, Bluetooth Classic and Bluetooth Low Energy) within a single chip, enabling on-board data processing while significantly reducing both complexity and physical size of the electronic board. In practice, the microcontroller manages the Bluetooth connection with the smartphone or PC, processes the acquired sensor data, and triggers the auditory stimulus. It also stores both raw sensor data and computed results on the on-board microSD (µSD) card. In the previous design, the SAMD21G18A microcontroller operated at a maximum clock frequency of 96 MHz, with 32 kB of RAM and 256 kB of program memory. This upgrade provides greater real-time computing capability without compromising measurement timing and allows the storage of large data vectors in RAM. Consequently, the new design supports a high sampling frequency of 1 kHz, previously limited to 200 Hz in the earlier prototype.
- Buzzer and LEDs: To generate the auditive stimulus, a custom-designed external electronic board was equipped with a centrally mounted buzzer and two LEDs situated on each side of the buzzer (see Figure 1 and Figure 2). These indicator LEDs are used to trigger a 240-fps HS camera at the onset of the buzzer sound, allowing comparison of reaction times measured by both systems, with the HS camera serving as the gold standard. In addition, a third fixed LED (the tracking LED) was included to track the movement with the HS camera, enabling precise detection of the subject’s motion. More details about the experimental setup are given in Section 3.
- Reduced power consumption: Power consumption was experimentally measured using a DC Power Analyzer (N6705A, Keysight Tech., Santa Clara, CA, USA) setting the output voltage at 3.7 V to simulate the supply of the lithium battery. When the device was connected via Bluetooth to the smartphone and the tracking LED was on, but without taking measurements, the current consumption was 54 mA, corresponding to a battery life of 8.3 h. In full-speed measurement mode, the current consumption increased to 117 mA, reducing the battery life to 3.84 h. Since the system operates in full-speed measurement mode only during the sprint tests, the battery capacity is sufficient to support measurement sessions in study participants or during personal use.

### 2.2. Firmware Description

The firmware was developed to optimize processor resources and to prevent saturation of either the Bluetooth communication channel or the µSD card writing process, given that measurements are performed at very high frequency. The execution sequence is as follows:

1. Upon system initialization, the tracking LED turns on, the Bluetooth device is paired, and the system enters a waiting state for the start command.
2. Stack-allocated acceleration arrays are created, reserving space in the processor’s RAM (Random Access Memory) for high-frequency data recording. This memory is released once the main function execution is completed. A random time seed is also generated.
3. 0.5 s before the random time, high-frequency recording (1 kHz) of the three acceleration components begins, while the acceleration magnitude is simultaneously calculated and stored in a separate array.
4. When the random time is reached, the system turns on the indicator LEDs and triggers the buzzer for 500 ms to provide the auditory cue.
5. 3.3 s after the random time, the system stops recording, turns off the indicator LEDs, and begins post-processing the data, ensuring that all measurements fit within the allocated acceleration arrays.
6. The acceleration magnitude array is processed. If the acceleration exceeds the desired trigger before the buzzer sounds, or if the acceleration does not reach the trigger after the buzzer, an error is indicated by blinking the indicator LEDs. In such cases, the data are neither saved to the µSD card nor transmitted via Bluetooth.
7. If the data are valid, both the raw data and the processed results are saved to the µSD card and transmitted via Bluetooth to the user’s smartphone.

**Figure 3:**
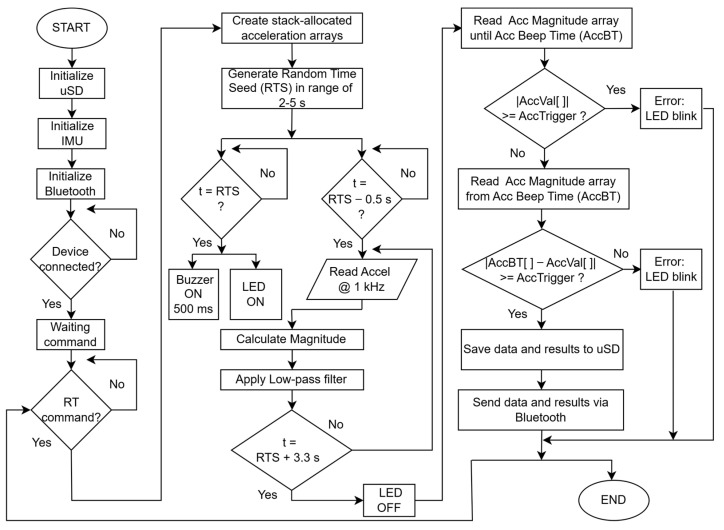
Firmware flowchart. Abbreviations: µSD: microSD card; RT: Reaction Time; RTS: Random Time Seed; AccBT: Accelerometer Beep Time; AccVal: Acceleration Value; INDLED: Indication LED; TRKLED: Tracking LED.

This process is illustrated in the flowchart presented in Figure 3. It is important to highlight that the Bluetooth latency has no impact in the RT acquisition process, since the latency of the communication could only affect the previous random time when the sound stimulus is emitted. When the sprint test has finished, the results are immediately transmitted to the user’s smartphone, so the Bluetooth latency does not play any role in the RT measurement process.

### 2.3. Desktop Application

**Figure 4:**
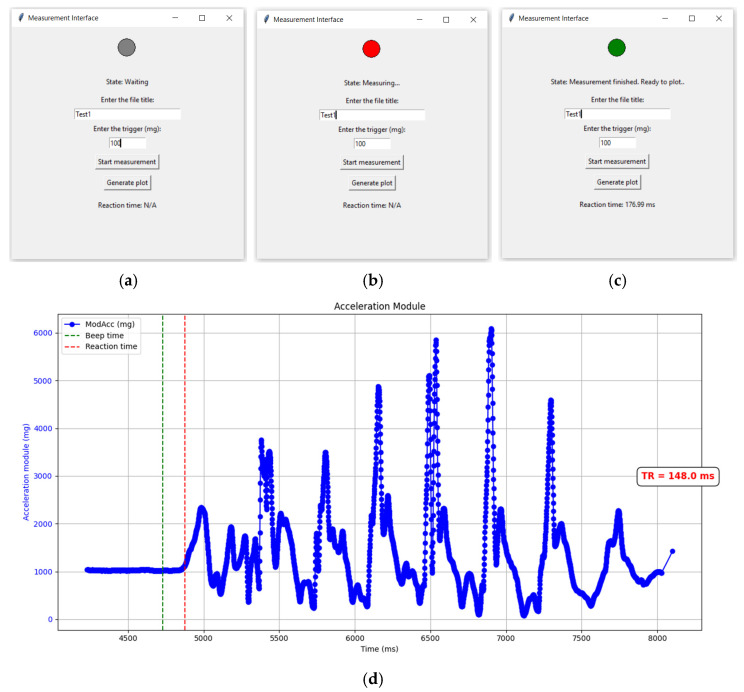
(**a**–**c**) Desktop application states and output plots; (**d**) Plot of the acceleration magnitude automatically generated by the desktop application.

A desktop-based application was developed to control the IMU, as well as to record and visualize acceleration data. The connection to the device is established via Bluetooth Classic, a widely used and reliable protocol supported by most computers. The acquired acceleration data are automatically stored in a plain text file within a dedicated folder created by the program. An intuitive interface was implemented to guide the user through three system states, indicated by the color of a bulb icon (see Figure 4a–c). In the initial state (gray), the user can specify a new file name, select the desired trigger, or generate plots from previous tests. When the test is running (red), the IMU performs the measurement and sends the processed data to the PC once completed. In the final state (green), the reaction time is displayed, and the user may either initiate a new test or generate plots from the current or previous sessions. For end users, it is not necessary to set the trigger value after the initial calibrations, since it has been predefined to yield a reaction time closely aligned with the gold standard (HS camera). This is described in detail in the Results section. Initially, this application was also employed to acquire data and perform the calibration procedure.

When a file is selected and the Generate plot button is pressed, the program automatically generates a plot of the acceleration magnitude (Figure 4d). In the plot, the beep onset is marked by a vertical green dotted line, while the reaction time is indicated by a vertical red dotted line. The corresponding reaction time value is also shown on the right-hand side of the graph. The dedicated software was developed using Visual Studio Code 1.102.3 and Python 3.13 (64-bit). The device employed for testing was an Asus Tuf Gaming F15.

### 2.4. Smartphone Application

A smartphone application was developed to control the system, acquire data, and provide an intuitive interface that delivers instant user feedback. This mobile solution offers a simpler and more accessible alternative to a PC-based application, especially for use in on-field settings. The device connects to the application via Bluetooth Classic using the Serial Port Profile (SPP), ensuring stable wireless communication and enabling full management of system functionalities. The interface features a quick-control menu with two buttons to start and end the test, along with a status indicator confirming successful device connection. It also integrates a circular gauge that displays reaction times after each test, using a color-coded scale to highlight performance relative to response speed. In addition, a dedicated panel and text box are provided to configure and manage the complete set of system functionalities (see Figure 1). The application was implemented in Android Studio (version 2020.3.1) and programmed for compatibility with Android 12.0 (API level 31). Validation tests were conducted using a Xiaomi Redmi Note 9 smartphone (Xiaomi Inc., Beijing, China).

## 3. Experimental Setup and Methods

Five healthy male athletes from the University of Cádiz (Spain) voluntarily participated in the study. The participants were selected to ensure heterogeneity—particularly in terms of age, which is known to strongly influence reaction time—to include a range of cases and verify that the system performs reliably across different user profiles. The participants’ ages ranged from 26 to 59 years, with a mean age of 47.0 ± 15.4 years. The mean height and body mass were 1.79 ± 0.06 m and 77.4 ± 7.0 kg, respectively. These characteristics correspond to a mean body mass index (BMI) of 24.2 ± 1.6 kg·m^−2^. Thanks to the heterogeneity of the subjects under test, the obtained reaction times ranged from 33 ms to 270 ms, according to the commercial HS camera-based system. The participants performed the sprint start tests at the athletics track of the Bahía Sur Sports Complex (Cádiz, Spain). The tests consisted of performing 5 sprint starts per participant simulating a competitive event (Figure 1). Prior to the tests, they underwent a 15 min warm-up that included light running, vertical jumps, and stretching. Finally, the subjects performed the tests.

Participants begin in a standardized crouched sprint-start position, as shown in Figure 1. The inertial sensor is positioned at the lower back, centered approximately over the sacrum using an elastic belt to align with the body’s center of mass. This location ensures optimal capture of the initial trunk acceleration associated with the impulse phase of sprint initiation. Each trial begins with the subject holding the start position motionless. An auditory cue (4 kHz tone, 500 ms duration) is delivered through the buzzer attached to the electronic board. Upon hearing the cue, the participant initiates the sprint start as quickly as possible, driving explosively forward from the blocks.

The IMU continuously streams triaxial acceleration data to the smartphone. The system detects movement onset by identifying the first acceleration peak exceeding a dynamic threshold relative to the resting baseline in the forward (anterior–posterior) axis. A typical threshold of ±0.1 g is used, with filtering to reject tremor or anticipatory motion. Reaction time is computed as the elapsed time between the auditory cue emission and the moment when the threshold is crossed. An explanation video can be found in the Supplementary Materials.

To ensure robust results, each subject performs 5 repetitions, with randomized inter-trial intervals between 2 and 5 s to prevent anticipation. The application displays reaction time values immediately and logs all trial data (timestamped cue onset, motion onset, raw acceleration trace, and repetition number) for further analysis. This protocol allows the system to capture full-body motor readiness and response speed under conditions that closely mimic actual athletic performance. Compared to traditional reaction time measurements, it provides a more integrated neuromechanical marker of response latency, especially relevant in sprinting, football, or start-dominant sports.

To validate the accuracy of the reaction time measurements obtained from the developed IMU-based system, a comparative analysis was performed using a high-speed video camera (Casio Exilim HS ZR1000 from Casio Computer Co., Ltd., Tokyo, Japan) operating at 240 fps. A small LED marker was affixed near the participant’s lower back, adjacent to the IMU housing. The camera was mounted on a Manfrotto tripod (Cassola, Italy), positioned perpendicular to the starting line at a distance of 5 m. Subsequently, the videos were analyzed using the kinematic analysis software Kinovea (version 2023.1). This software was used to quantify the duration, frame to frame, of the time elapsed from the moment the LED, integrated into the inertial system, turned on until the first observable displacement of the hip was detected. The HS camara can record up to 1000 fps. However, the image quality at this rate is too blurry to accurately determine the start of the movement; therefore, the authors considered the lower frame rate of 240 fps as the optimal configuration to minimize experimental error. In any case, the sampling frequency of 240 fps has been previously validated for velocity training studies [34] and for measuring flight time in vertical jumps [35], among other applications in sport science that require high-speed data sampling. These studies support 240 fps as a reliable method for time-based measurements. The LED provided a visually distinct point for motion tracking during post-processing. The timestamp of the auditory stimulus was synchronized with the video and the IMU data using a visual flash and an audio tone, ensuring sub-frame alignment across systems. This approach, which has been widely used in validation studies of wearable motion sensors, provides a reliable ground-truth reference for estimating movement onset and verifying IMU-based reaction time detection. The comparison confirmed a high level of temporal agreement between the IMU-detected onset and the video-based reference, with discrepancies consistently below the sampling resolution of the inertial system.

## 4. Results and Discussion

**Figure 5:**
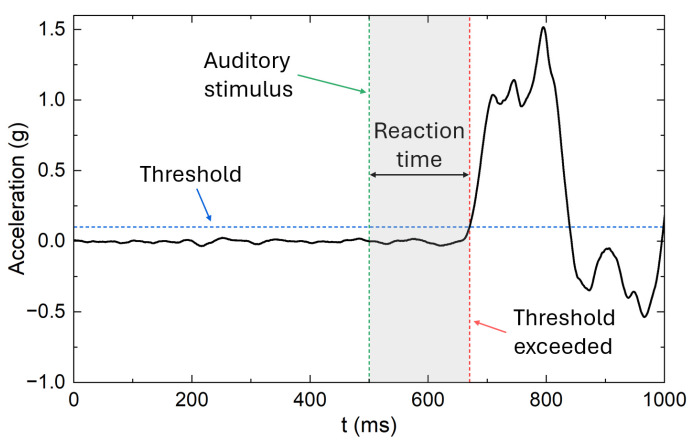
Example acceleration signal during a sprint start, showing auditory stimulus onset, reaction time, and movement initiation relative to the predefined threshold.

The IMU-based system successfully recorded high-frequency acceleration signals during all sprint start trials, enabling precise identification of both stimulus onset and subsequent motor response. Across participants, the pre-stimulus phase was characterized by stable baseline values, while the transition to movement onset was consistently marked by a clear suprathreshold increase in acceleration. This allowed reliable computation of reaction times in each repetition. Figure 5 illustrates a representative trial, highlighting the sequence of events used for reaction time detection. The auditory stimulus is marked by the green dashed line, followed by a short latency period during which the acceleration signal remained near baseline. Movement onset was identified when the signal crossed the predefined threshold (blue dashed line), corresponding to the beginning of the voluntary response. The reaction time, calculated as the interval between the stimulus and this suprathreshold event (gray-shaded area), was followed by a pronounced acceleration peak associated with the propulsion phase of the sprint start.

**Figure 6:**
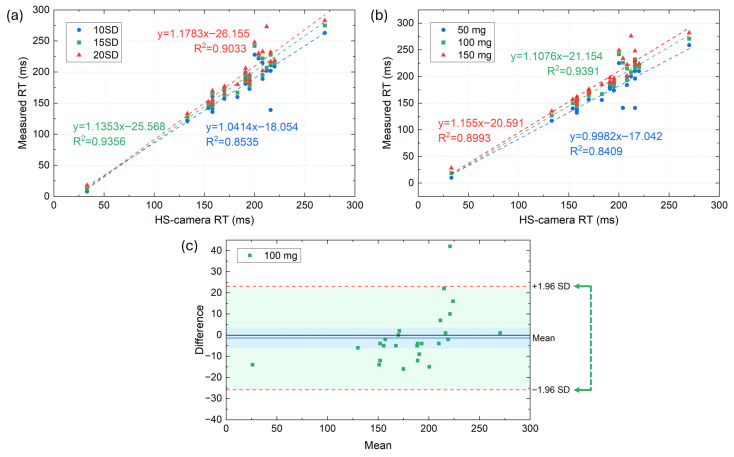
Relationship between IMU-derived and HS-camera reaction times for: (**a**) the dynamic thresholds of 10SD, 15SD, and 20SD; and (**b**) the fixed-magnitude thresholds (50 mg, 100 mg, and 150 mg); (**c**) Bland–Altman plot comparing IMU-derived and HS-camera reaction times using the fixed threshold of 100 mg.

To determine the acceleration threshold for reaction time detection, we initially evaluated a dynamic approach in which the detection limit was defined as a multiple of the baseline noise level, expressed as N times the standard deviation (SD) of the pre-stimulus acceleration signal. Three different scaling factors were tested (10SD, 15SD, and 20SD), and the estimated reaction times were compared against the reference values obtained from the HS video analysis. The results, depicted in Figure 6a, show a strong linear relationship between IMU-derived and HS-camera reaction times across all conditions, but the highest coefficient of determination was observed for the 15SD threshold, indicating superior temporal agreement and minimal systematic bias compared to both lower (10SD) and higher (20SD) thresholds. However, while the SD-based approach demonstrated good accuracy, its dependency on trial-specific baseline variability made its implementation less practical for real-time applications, as the effective threshold would vary across subjects and repetitions.

To enable a simpler and more robust implementation, we derived an equivalent fixed-magnitude threshold by averaging the acceleration values corresponding to the optimal 15SD dynamic threshold across all participants and trials, yielding an approximate value of 111.2 mg. Based on this result, we tested three fixed thresholds (50 mg, 100 mg, and 150 mg) and repeated the same comparative analysis against HS-camera measurements. Figure 6b shows that the fixed threshold of 100 mg provides the best trade-off between accuracy and robustness, achieving high linearity and minimizing both detection delays and noise-driven false positives. At this threshold, the correlation factor between the results obtained from the developed system and the HS camera was R^2^ = 0.9391, indicating good agreement between the data reported by both systems. In contrast, excessively low thresholds (50 mg) increased variability and susceptibility to baseline fluctuations, whereas higher thresholds (150 mg) systematically underestimated reaction times by delaying the detection of movement onset.

To further evaluate the agreement between the IMU-derived reaction times and the HS-camera reference at the selected 100 mg threshold, a Bland–Altman analysis was performed (Figure 6c). The results showed a mean bias of –1.35 ms (95% CI: –6.28 to 3.59 ms, blue band in Figure 6c), indicating a minimal and statistically nonsignificant tendency of the IMU system to slightly underestimate reaction times. Green band in Figure 6c depicts the 95% limits of agreement, which ranged from –25.8 to +23.1 ms (95% CI of limits: –34.4 to –17.3 ms for the lower bound, and 14.6 to 31.7 ms for the upper bound), encompassing nearly all paired observations. Importantly, no proportional bias was observed, as the differences were uniformly distributed across the range of measured reaction times, confirming that measurement accuracy remained stable for both shorter and longer latencies. Taken together, these results demonstrate that the proposed IMU-based system achieves good temporal agreement with the high-speed camera reference, with deviations well within the range acceptable for applied use in dynamic, full-body reaction time assessments.

For the selected threshold of 100 mg, a mean RT of 185 ± 47 ms was obtained across all sprint tests of the five participants. The maximum and minimum RT values were 271 ms and 19 ms, respectively. The lowest value should be considered invalid in a competition, as regulations for sprint starts specify that a start is considered false if the athlete moves within 100 ms of the starting gun’s sound [36]. However, for the purpose of system validation, the authors chose to retain this value in the analysis in order to expand the range of data considered. These results demonstrate that, although the sample size was limited, the heterogeneity of the participants enables the validation of the system across a wide range of reaction times.

### Strengths and Limitations

The main distinguishing feature of the developed system for measuring reaction time in athletic starts lies in its high sampling frequency, which enables highly accurate RT acquisition. In addition, the system is considerably simpler, more portable, and more affordable than other well-known RT measurement solutions, such as camera-based systems. From a technical perspective, this system provides RT measurement independently of the Bluetooth latency or any synchronization errors.

However, the system has presents certain drawbacks and limitations that must be acknowledged. First, the placement of the sensor (i.e., attached to the athlete’s body) may have a potential impact on or interfere with the athletes’ movements, thereby potentially affecting performance. Although this issue is inherent to the wearable nature of the device, it has been specifically designed to be lightweight and portable to minimize any possible impact. Nevertheless, this aspect should be carefully considered in high-level competitions. Another limitation of the developed system is the maximum distance it can be separated from the smartphone, which is constrained by the Bluetooth Classic communication range, typically around 10–20 m.

Regarding the limitations of the study itself, it should be noted that the sample size was limited to five subjects. Although this number was sufficient to demonstrate the functionality of the prototype and validate the proposed proof of concept, a more extensive study involving a larger sample size would be required to achieve a higher Technology Readiness Level (TRL 7–8). Nevertheless, the five participants were intentionally selected to ensure heterogeneity (particularly in terms of age, which is known to strongly influence reaction time) in order to include a range of cases and verify that the system performs reliably across different user profiles.

The developed system could be extended to handle multiple measurement units, which would enable the simultaneous recording of reaction times for multiple athletes. To support this expansion, future versions of the prototype should make use of Bluetooth Low Energy (BLE) rather than Bluetooth Classic, as BLE would allow easier and more precise wireless synchronization when multiple devices need to be coordinated, or when real-time transmission of measured data via BT is required. This capability would be particularly valuable, for instance, in high-level competition settings.

## 5. Conclusions

This work presented and validated a portable IMU-based system for measuring reaction time during sprint starts. By integrating high-frequency acceleration acquisition, synchronized auditory stimulation, and a fixed threshold calibrated against a high-speed camera, this system demonstrated high accuracy and good agreement with the reference method. Compared with existing approaches, it offers a field-ready, low-cost solution that captures the onset of whole-body movement with high temporal resolution, making it suitable for both sports’ performance monitoring and research applications. While the results confirm the feasibility and robustness of the approach, future work should extend validation to larger populations, explore different sensor placements, and assess applicability in clinical contexts where reaction time is a relevant functional marker.

## Data Availability

The data presented in this study are available on request from the corresponding author.

## Supplementary Materials

The following supporting information can be downloaded at https://www.mdpi.com/article/10.3390/s25216730/s1: Video S1: Video_IMU_RT_Sensors.mp4.

## Abbreviations

The following abbreviations are used in this manuscript:

RT	Reaction Time
IMU	Inertial Measurement Unit
BT	Bluetooth
